# Neurocognitive and observational markers: prediction of autism spectrum disorder from infancy to mid-childhood

**DOI:** 10.1186/s13229-017-0167-3

**Published:** 2017-09-22

**Authors:** Rachael Bedford, Teodora Gliga, Elizabeth Shephard, Mayada Elsabbagh, Andrew Pickles, Tony Charman, Mark H. Johnson

**Affiliations:** 1 Biostatistics Department, Institute of Psychiatry, Psychology & Neuroscience, King’s College London, London, UK; 2 Centre for Brain and Cognitive Development, Birkbeck College, University of London, London, UK; 3 MRC Social, Genetic & Developmental Psychiatry Centre, Institute of Psychiatry, Psychology & Neuroscience, King’s College London, London, UK; 4 Department of Psychiatry, McGill University, Montreal, Canada; 5 Psychology Department, Institute of Psychiatry, Psychology & Neuroscience, King’s College London, London, UK

**Keywords:** Autism, High risk, Infants, Siblings, Diagnosis, Prediction, Neurocognitive

## Abstract

**Background:**

Prospective studies of infants at high familial risk for autism spectrum disorder (ASD) have identified a number of putative early markers that are associated with ASD outcome at 3 years of age. However, some diagnostic changes occur between toddlerhood and mid-childhood, which raises the question of whether infant markers remain associated with diagnosis into mid-childhood.

**Methods:**

First, we tested whether infant neurocognitive markers (7-month neural response to eye gaze shifts and 14-month visual disengagement latencies) as well as an observational marker of emerging ASD behaviours (the Autism Observation Scale for Infants; AOSI) predicted ASD outcome in high-risk (HR) 7-year-olds with and without an ASD diagnosis (HR-ASD and HR-No ASD) and low risk (LR) controls. Second, we tested whether the neurocognitive markers offer predictive power over and above the AOSI.

**Results:**

Both neurocognitive markers distinguished children with an ASD diagnosis at 7 years of age from those in the HR-No ASD and LR groups. Exploratory analysis suggested that neurocognitive markers may further differentiate stable versus lost/late diagnosis across the 3 to 7 year period, which will need to be tested in larger samples. At both 7 and 14 months, combining the neurocognitive marker with the AOSI offered a significantly improved model fit over the AOSI alone.

**Conclusions:**

Infant neurocognitive markers relate to ASD in mid-childhood, improving predictive power over and above an early observational marker. The findings have implications for understanding the neurodevelopmental mechanisms that lead from risk to disorder and for identification of potential targets of pre-emptive intervention.

**Electronic supplementary material:**

The online version of this article (10.1186/s13229-017-0167-3) contains supplementary material, which is available to authorized users.

## Background

A key criticism of psychiatric diagnoses based on behavioural criteria is that, unlike diagnoses elsewhere in medicine, they lack a defined biological basis. Consequently, some have argued for a more biologically based approach within psychiatry based on phenotypes described in terms of neurocognitive measures associated to specific brain systems or pathways [1, 2]. In autism spectrum disorder (ASD), a number of neurocognitive phenotypes [3] have been identified during childhood that are associated with the core ASD symptom domains of social communication impairments, the presence of restricted and repetitive patterns of behaviour and atypical sensory responses (*Diagnostic and Statistical Manual 5th edition* (DSM-5) [4]). For early emerging neurodevelopmental disorders such as ASD, there has been an increasing emphasis on identifying earlier neurocognitive phenotypes, that might be evident before the onset of frank clinical symptoms, and which are referred to as ‘antecedents’ [5]. In the past decade, the use of a prospective familial high-risk design, with a recurrence rate close to 20% [6, 7], has enabled the discovery of a variety of antecedents of later ASD symptoms from the first few months of life. Indices of infant behavioural and neural atypicalities, both measured through observational scales and specific infant neurocognitive markers, as well as various neuroimaging methods, have been shown to associate with a later ASD diagnosis in toddlerhood (for reviews, see [8–10]). Observational scales and neurocognitive measures use different approaches to measuring psychopathology. The former aims to measure the extent of atypicality by counting the occurrence of a broad spectrum of behaviours; for example, the Autism Observation Scale for Infants (AOSI [11]) assesses behaviours such as the anticipation of social interaction, imitation and motor skills. Total scores measured when infants at risk for ASD are 1 year old predict later ASD symptoms and diagnostic outcome [12, 13]. In contrast, neurocognitive measures are designed to index the functioning of particular cognitive or neural systems. The British Study for Infant Siblings (BASIS) has published on several such neurocognitive markers [10]. Two of those that showed an association with 3-year-old ASD diagnosis are reduced differentiation in the neural response to eye gaze shifts towards versus away from the infant at 7 months of age, measured by the amplitude of the P400 event-related potential (ERP) component [14] and prolonged attention disengagement latency at 14 months of age, measured in a visual orienting task [13, 15, 16].

The extent to which such antecedents are predictive markers for diagnosis beyond early childhood is, as yet, unexplored, with most studies reporting prediction of 2 and 3 year diagnosis. As with other behaviourally defined disorders [17], it is known that there is change in individual trajectories of ASD manifestation over time, particularly in early childhood [18–21]. In different children, symptoms can ease or worsen to the extent that children move above and below specific diagnostic thresholds [22–24]. The only two high-risk sibling studies that have reported to date on stability of diagnosis to mid-childhood [25, 26] both found overall good agreement in clinical best-estimate diagnosis of ASD between 3 years and mid-childhood. However, in both studies, the diagnosis was not entirely stable. Brian et al. [25] found one individual out of 18 classified as ASD at 3 years of age no longer met criteria in middle childhood (9 years), and 6 high-risk children out of 49 who were not considered to meet criteria for ASD at 3 years went on to be later diagnosed. A similar pattern emerged in our mid-childhood follow-up of the present sample at age 7 years [26], where 3 out of 13 children considered to meet ASD diagnostic criteria at 3 years did not at the mid-childhood assessment, and 5 out of 29 children who were not considered to meet ASD criteria at 3 years did so at 7 years. The latter pattern is consistent with the notion, included in DSM-5, that in some children with ASD, symptoms might not become apparent until ‘demands exceed capacity’ [4]. This is consistent with our clinical experience that for some children—often those with high general ability, good language and relatively intact non-verbal communication skills—it is only when they enter preschool, where they are faced with increased expectations of behaviour, in particular their responses to peers and unfamiliar adults, that parents, carers and other professionals become concerned about possible atypical development.

Given the diagnostic change from early childhood, the question arises as to whether markers remain predictive of ASD diagnosis into mid-childhood. In the current study, we first aim to test whether two previously reported infant neurocognitive markers, indexing eye-gaze processing [14] and attention control [15] and one observational scale of early emerging behavioural atypicalities (the AOSI) [12], shown to be associated with ASD outcome at 3 years of age, also distinguish high risk (HR) children with and without a diagnosis of ASD at age 7 years. To our knowledge, this is the first report of an association between infant neurocognitive markers and mid-childhood ASD diagnosis. Our second aim is to establish how the markers work in combination, by testing whether inclusion of the neurocognitive marker (i.e. P400 in response to gaze shifts or disengagement) in the same model as the AOSI (measured concurrently with the neurocognitive marker, i.e. 7 or 14 months) improved prediction of ASD outcome over the AOSI alone.

## Methods

### Participants

As part of the British Autism Study of Infant Siblings (BASIS: http://basisnetwork.org/), one hundred and four infants (54 high risk, 21 male; 50 low risk, 21 male) took part in a battery of assessments at 7 and 14 months and 2, 3 and 7 years. At enrolment, each high risk (HR) infant (*n* = 54) had an older sibling (in 4 cases, a half-sibling) with a community clinical ASD diagnosis, confirmed using information from the *Development and Well-Being Assessment* (*DAWBA* [27]) and the *Social Communication Questionnaire* (*SCQ* [28]) by expert clinicians on our team (TC, PB).^1^ Low risk (LR) controls (*n* = 50) were full-term infants (with one exception) recruited from a volunteer database at the Birkbeck Centre for Brain and Cognitive Development. For older siblings of LR infants, the SCQ was used to confirm absence of ASD, with no child scoring above instrument cut-off (≥ 15; *n* = 1 missing data).

Of 53 HR and 48 LR children retained at the 3-year assessment, 44 HR (83%) and 37 LR (77%) agreed to take part in the follow-up study at 6–8 years. Of these, two HR children did not complete a research visit (parents completed questionnaires only). As we did not see these children, we were unable to assign them to an ASD outcome group and consequently excluded them from the current analyses, leaving a final sample of 42 HR siblings (15 boys, 27 girls) and 37 LR controls (15 boys, 22 girls). The retained sample did not differ from the non-retained sample in 3 year levels of ASD on the *Autism Diagnostic Observation Schedule—Generic* (ADOS-G [29]), *Social Responsiveness Scale—Second Edition* (*SRS-2* [30]) or SCQ, developmental level on the *Mullen Scales of Early Learning* (*MSEL* [31]), adaptive behaviour assessed with the *Vineland Adaptive Behavior Scales—Second Edition* (*VABS-II* [32]), or family income (all *p* > 0.4). The HR and LR groups did not differ in age (HR mean (SD) 90.6 (6.3) months; LR mean (SD) 89.3 (4.9) months; *t*(74) = − 1.00, *p* = 0.31) or sex (HR % male 35.7; LR % male 40.5; *Ӽ* ^2^ (1) = .20, *p* = 0.66) at the follow-up. Ethical approval was obtained from the NHS National Research Ethics Service (NHS RES London REC 08/H0718/76; 14/LO/0170). Parents provided written informed consent. At the mid-childhood visit, children provided written informed assent wherever possible given developmental level.

### Procedure

The association between infant neurocognitive markers (neural gaze processing and disengagement latencies) as well as the AOSI scores and 3 year ASD outcomes has been previously reported (see [12, 14, 15] for full description of the methods). See Additional file 1 for details of our other previously published antecedent biomarkers.

#### Gaze processing task: P400 difference score (see [14])

In this task, infants saw images of four female faces with gaze directed either towards or away from the infant. Each trial block began with a static colourful fixation stimulus followed by three to six face stimuli (same identity) alternating between direct gaze and averted gaze. This gave the impression of gaze shifts away and towards the infant. Faces were aligned with the centre of the screen with the eyes appearing at the same location as the fixation stimuli, to ensure that infants were fixating the eye region. The faces subtended 21 × 14 degrees of visual angle. Fixation stimuli, preceding the onset of the face and noise stimuli, subtended approximately 1.6 × 1.6 degrees and were presented for a variable duration of 800 to 1200 ms. Each trial lasted for 1000 ms.

Infants wore a 128 channel Hydrocel Sensor Net on their head and were seated on the parent’s lap in front of the stimulus screen. When the infant was attending to the screen, trials were presented continuously for as long as the infant remained attentive, with brain electrical activity measured simultaneously using the vertex as a reference (Cz in the conventional 10/20 system). EGI NetAmps 200 was used (gain = 1000). Data were digitised with a sampling rate of 500 Hz and band-pass filtered between 0.1 and 100 Hz. Segmented data were processed using standard procedures including interpolation of missing channels, re-referencing to the average reference and baseline correction. Subsequent to artefact rejection, ERP components were ascertained based on visual inspection of grand averages. Three task-sensitive components (P100 120–199 ms, N290 200–319 ms and P400 320–520 ms) were ascertained over the occipito-temporal region, and their characteristic amplitude and latency were computed for each infant. Elsabbagh and colleagues [15] reported on P400 differences in relationship to 3-year-old diagnosis, with infants who went on to develop ASD showing less discrimination of away versus towards gaze shifts. We therefore focus on the same component. There were no significant outcome group differences (LR, HR-No ASD, HR-ASD) in the number of trials included in the analysis of the P400, *F*(2, 69) = 1.84, *p* = 0.17.

#### Attention Disengagement (see [15])

Infants were presented with the stimuli on a 46″ LCD monitor, while seated on their parent’s lap at 60cm distance. Looking behaviour was recorded with a video camera, and trial presentation was controlled by the experimenter. Each trial began with a central stimulus (subtending 13.8° × 18.0°) followed by a peripheral target green balloon (subtending 6.3° × 6.3°) which appeared randomly on the left or right. The target remained on the screen until either (1) the infant looked at it or (2) the maximum time of 2.5 s passed. An animal reward stimulus (elephant, lion, seal etc.) then appeared in the place of the green balloon. Up to 70 trials were presented depending on infants’ attentiveness. There were two trial types in this study: baseline and overlap. In the baseline condition, the central stimulus disappeared at the *same time* as the peripheral target appeared, whereas in the overlap condition, the central stimulus remained present while the target stimulus appeared in the periphery. Elsabbagh and colleagues [15] reported on disengagement (the saccadic reaction time difference between overlap and baseline trials) in relation to 3-year-old diagnosis, with infants who later developed ASD showing the longest disengagement latencies. We therefore focus on the same measure in this paper. The number of valid trials did not differ by outcome group: *F*(2, 72) = 1.02, *p* = 0.37 (see Table 1).

*The Autism Observation Scale for Infants* (AOSI [11, 12]) is a semi-structured observational assessment of ASD behavioural markers in infancy collected at 7 and 14 months. In the current study, a 19-item version of the AOSI was used (see [33]) which gives a total score (sum of all codes; max score 44), with higher scores indexing greater atypicality. The majority of assessments were double coded with excellent reliability (*n* = 85, intraclass correlation coefficient = 0.95).

### ASD outcome at 7 years of age

To ascertain ASD diagnostic outcome according to DSM-5, four experienced researchers (ES, BM, GP, TC) reviewed information on ASD symptomatology (ADOS-2, *Autism Diagnostic Interview—Revised*, ADI-R [34] and SCQ; for HR participants only) and adaptive functioning (VABS-II) and IQ (*Wechsler Abbreviated Scale of Intelligence—Second Edition*, *WASI-II* [35]) for each HR and LR child. Clinicians were not involved in the infant visits when the experimental tasks were administered. Diagnosis at age 7 years included review of all information previously obtained, and there was overlap in the personnel involved in the diagnostic decision-making (GP, TC). However, the age 7 diagnostic decisions were not directly yoked to the diagnostic decisions previously taken at the 3-year visit but an independent decision was made as to whether, based on all the clinical information collected at the 2-, 3- and 7-year assessments, the child currently met DSM-5 criteria for ASD. Fifteen HR children (7 boys, 8 girls) met DSM-5 (APA, 2013) criteria for ASD at age 7, and the remaining 27 HR children (8 boys, 19 girls) did not. Of 13 HR siblings with an ASD diagnosis at 3 years who were also seen at 7 years, 10 retained their ASD diagnosis (76.9%, ‘stable diagnosis’) and 3 did not (23.1%, ‘lost diagnosis’). Of the 29 HR who were not given an ASD diagnosis at 3 years also seen at 7 years, 24 did not meet diagnostic criteria for ASD (82.8%) at 7 years but 5 did meet ASD criteria (17.2%, ‘late diagnosed’). The 15 HR children meeting DSM-5 criteria for ASD at age 7, including the 5 ‘later diagnosed’ children, formed the HR-ASD group. Of the 27 HR children who did not meet ASD criteria at age 7, the 3 ‘lost diagnosis’ children were excluded from further analysis (given they met ICD-10 criteria [36] for ASD earlier in development), leaving 24 HR children in the HR-No ASD group (see Additional file 1 for more information on the groups split by diagnostic change). None of the 37 LR children met DSM-5 criteria for ASD, and none had a community clinical ASD diagnosis at 3 years nor at 7 years. Group characteristics at age 7 are presented in Table 2.

**Table 2:** Descriptive statistics from the 7-year visit RB

	Low risk	High-risk No ASD	High-risk ASD	Group differences
Age (months)				
Mean (SD)	89.34 (4.81)	91.42 (6.28)	89.13 (6.53)	n/s
*N* (girls)	35* (21)	24 (19)	15 (8)	
ADI-social				
Mean (SD)	Not completed	4.04 (5.48)	13.14 (4.69)	*t*(36) = − 5.20, *p* < 0.001
*N* (girls)		24 (19)	14 (8)	
ADI communication				
Mean (SD)	Not completed	4.25 (4.67)	10.43 (4.59)	*t*(36) = − 3.96, *p* < 0.001
*N* (girls)		24 (19)	14 (8)	
ADI RRB				
Mean (SD)	Not completed	0.58 (1.41)	3.57 (1.74)	*t*(36) = − 5.77, *p* < 0.001
*N* (girls)		24 (19)	14 (8)	
ADOS CSS total				
Mean (SD)	1.70 (1.19)	2.46 (1.41)	6.20 (2.78)	*F*(2, 69) = 37.35, *p* < 0.001, *η* ^2^ = 0.520
*N* (girls)	33 (19)	24 (19)	15 (8)	
ADOS CSS SA				
Mean (SD)	2.18 (1.70)	2.96 (1.60)	6.60 (2.59)	*F*(2, 69) = 29.11, *p* < 0.001, *η* ^2^ = 0.458
*N* (girls)	33 (19)	24 (19)	15 (8)	
ADOS CSS RRB				
Mean (SD)	1.12 (0.70)	3.04 (2.84)	6.13 (2.70)	*F*(2, 69) = 29.80, *p* < 0.001, *η* ^2^ = 0.463
*N* (girls)	33 (19)	24 (19)	15 (8)	
WASI FSIQ				
Mean (SD)	117.06 (11.61)	107.96 (12.76)	109.79 (21.36)	*F*(2, 70) = 3.25, *p* = 0.05, *η* ^2^ = 0.085
*N* (girls)	35 (21)	24 (19)	14 (8)	

*Two LR children completed questionnaires only; an exact age at visit was not available for these children

*ADI* Autism Diagnostic Interview—Revised, *RRB* Repetitive and restrictive behaviour, *ADOS* Autism Diagnostic Observation Schedule, *CSS* Calibrated severity score, *SA* Social affect, *WASI-II* Wechsler Abbreviated Scale of Intelligence—II Full-Scale IQ, *n/s* non-significant

**Table 1:** Neurocognitive markers and Autism Observation Scale for Infants at 7 and 14 months

		Low risk	High-risk No ASD	High-risk ASD
Visit 1	P400			
7 months	Valid trial no.	57.02 (32.61)	69.61 (32.15)	50.18 (30.24)
	Mean (SD)	3.11 (4.02)	1.63 (3.37)	− 2.02 (6.78)
	*N* (girls)	33 (20)	23 (18)	14 (8)
	AOSI			
	Mean (SD)	6.70 (3.67)	9.25 (5.14)	8.80 (7.39)
	*N* (girls)	37 (22)	24 (19)	15 (8)
	Mullen ELC			
	Mean (SD)	102.86 (10.76)	95.83 (9.88)	95.40 (18.39)
	*N* (girls)	37 (22)	24 (19)	15 (8)
	Age (months)	7.30 (1.18)	7.46 (1.22)	7.47 (1.36)
Visit 2	Disengagement			
14 months	Valid trial no.	27.03 (6.64)	26.86 (7.78)	24.20 (4.86)
	Mean (SD)	144.19 (102.88)	125.04 (135.13)	237.05 (122.73)
	*N* (girls)	36 (22)	22 (17)	15 (8)
	AOSI			
	Mean (SD)	3.31 (3.57)	3.30 (3.20)	7.53 (5.45)
	*N* (girls)	37 (22)	23 (18)	15 (8)
	Mullen ELC			
	Mean (SD)	108.46 (15.28)	104.48 (11.74)	93.33 (16.29)
	*N* (girls)	35 (22)	23 (18)	15 (8)
	Age (months)	13.72 (1.26)	13.74 (1.45)	13.80 (1.94)

*AOSI* Autism Observation Scale for Infants, *ELC* Early Learning Composite

### Statistical analysis

Analyses were run using SPSS [37] and Stata [38]. First, to assess the association between neurocognitive markers and ASD outcome in mid-childhood, separate multinomial logistic regressions were run, with 7-month AOSI and P400, and 14-month AOSI and disengagement latency, respectively, as predictors. For ASD outcome, the reference category was the HR-ASD group, with pairwise comparisons presented for LR versus HR-ASD, and HR-No ASD versus HR-ASD. In follow-up models, sex was entered as a factor but it did not impact the contribution infant markers made to predicting outcome (see Additional file 1). Next, to enable a direct comparison of the neurocognitive markers with observational markers of early ASD behaviours, the AOSI total score at 7 and 14 months and neurocognitive markers (P400 and disengagement latency, respectively) were included as predictors in the same logistic regression model, one for 7 month predictors and one for 14 month predictors. The chi-squared model fit for the model with each neurocognitive marker together with AOSI was then compared to that with AOSI or the neurocognitive marker alone.

## Results

### Association between infant markers and ASD outcome

When measured at 7 months, the AOSI total score was not a significant predictor of ASD outcome *χ* ^2^(2) = 4.40, *p* = 0.11. At 14 months, however, AOSI score did significantly predict ASD outcome *χ* ^2^(2) = 10.64, *p* = 0.005, with the HR-ASD group showing higher AOSI scores than LR controls (*B* = − 0.215, *SE* = 0.080, odds ratio = 0.81, *p* = 0.007) and HR-No ASD (*B* = − 0.215, *SE* = 0.089, odds ratio = 0.81, *p* = 0.016).

**Fig. 1:**
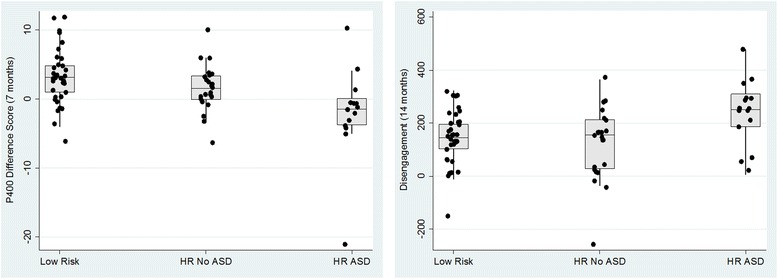
**a** P400 difference score at 7 months by 7-year-old ASD outcome. **b** Disengagement latency at 14 months by 7-year-old ASD outcome

**Fig. 2:**
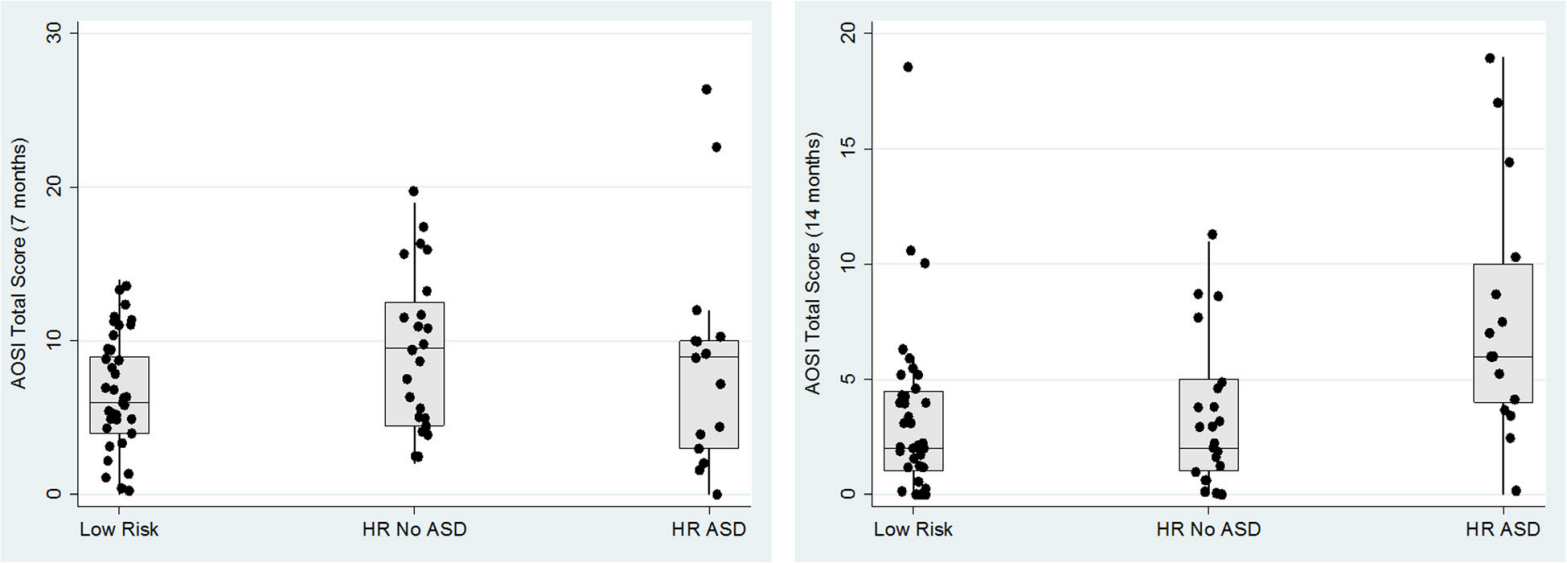
**a** AOSI Total Score at 7 months by 7-year-old ASD outcome. **b** AOSI Total Score at 14 months by 7-year-old ASD outcome

Seven-month P400 amplitude difference score was a significant overall predictor of the ASD outcome (*χ* ^*2*^(2) = 12.15, *p* = 0.002; see Fig. 1a), with the HR-ASD group differentiating less between the away and towards gaze shifts than LR controls did (*B* = 0.288, *SE* = 0.10, odds ratio = 1.33, *p* = 0.005). HR-ASD and HR-No ASD groups differed only marginally (*B* = 0.187, *SE* = 0.099, odds ratio = 1.21, *p* = 0.059). Results were substantively similar when one outlier was trimmed (see Additional file 1). Disengagement latency at 14 months also predicted overall ASD outcome (*χ* ^*2*^(2) = 9.55, *p* = 0.008; see Fig. 1b), with the HR-ASD group having significantly longer latencies than both other groups (LR: *B* = − 0.008, *SE* = 0.003, odds ratio = 0.99, *p* = 0.015; HR-No ASD: *B* = − 0.009, *SE* = 0.004, odds ratio = 0.99, *p* = 0.008) (Fig. 2).

### Prediction of neurocognitive and observational markers combined

In the combined model at 7 months, the omnibus test was significant *χ* ^2^(4) = 14.97, *p* = 0.005, with P400 (*χ* ^2^(2) = 10.54, *p* = 0.005 but not AOSI (*χ* ^2^(2) = 2.82, *p* = 0.24) predicting overall ASD outcome groups. The P400 discriminated the HR-ASD group from LR controls (*B* = 0.271, *SE* = 0.10, odds ratio = 1.31, *p* = 0.008) but only marginally from HR-No ASD (*B* = 0.191, *SE* = 0.10, odds ratio = 1.21, *p* = 0.057). The model containing both P400 and 7-month AOSI as predictors had a significantly better chi-squared model fit compared to the AOSI alone *χ* ^2^(2) = 10.54, *p* = 0.005 but was not significantly better than P400 score alone *χ* ^2^(2) = 2.82, *p* = 0.24.

The omnibus test for the combined model at 14 months was significant *χ* ^2^(4) = 18.31, *p* = 0.001. Both the AOSI (*χ* ^2^(2) = 8.76, *p* = 0.013) and disengagement latency significantly predicted ASD outcome (*χ* ^2^(2) = 6.56, *p* = 0.038), discriminating HR-ASD from both other outcome groups (AOSI: LR: *B* = − 0.206, *SE* = 0.086, odds ratio = 0.81, *p* = 0.017; HR-No ASD: *B* = −0.244, *SE* = 0.11, odds ratio = 0.78, *p* = 0.021; Disengagement: LR: *B* = − 0.007, *SE* = 0.004, odds ratio = 0.99, *p* = 0.04; HR-No ASD: *B* = − 0.009, *SE* = 0.004, odds ratio = 0.99, *p* = 0.023). The combined disengagement and AOSI at 14 months improved chi-squared model fit by *χ* ^2^(2) = 6.56, *p* = 0.04 compared to AOSI alone and by *χ* ^2^(2) = 8.76, *p* = 0.01 compared to disengagement alone.

### Analysis taking into account the changes in diagnosis between 3 and 7 years of age

**Fig. 3:**
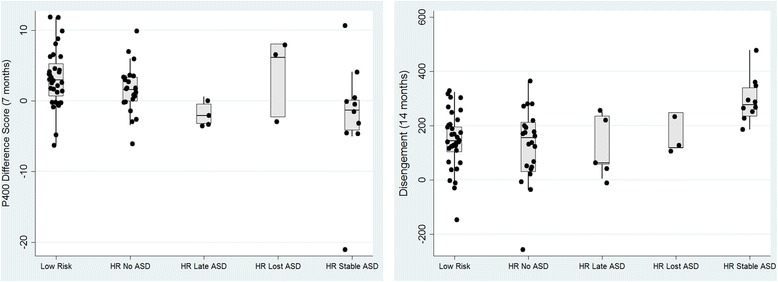
**a** P400 difference score at 7 months by stability of ASD outcome between 3 and 7 years. **b** Disengagement latency at 14 months by stability of ASD outcome between 3 and 7 years

**Fig. 4:**
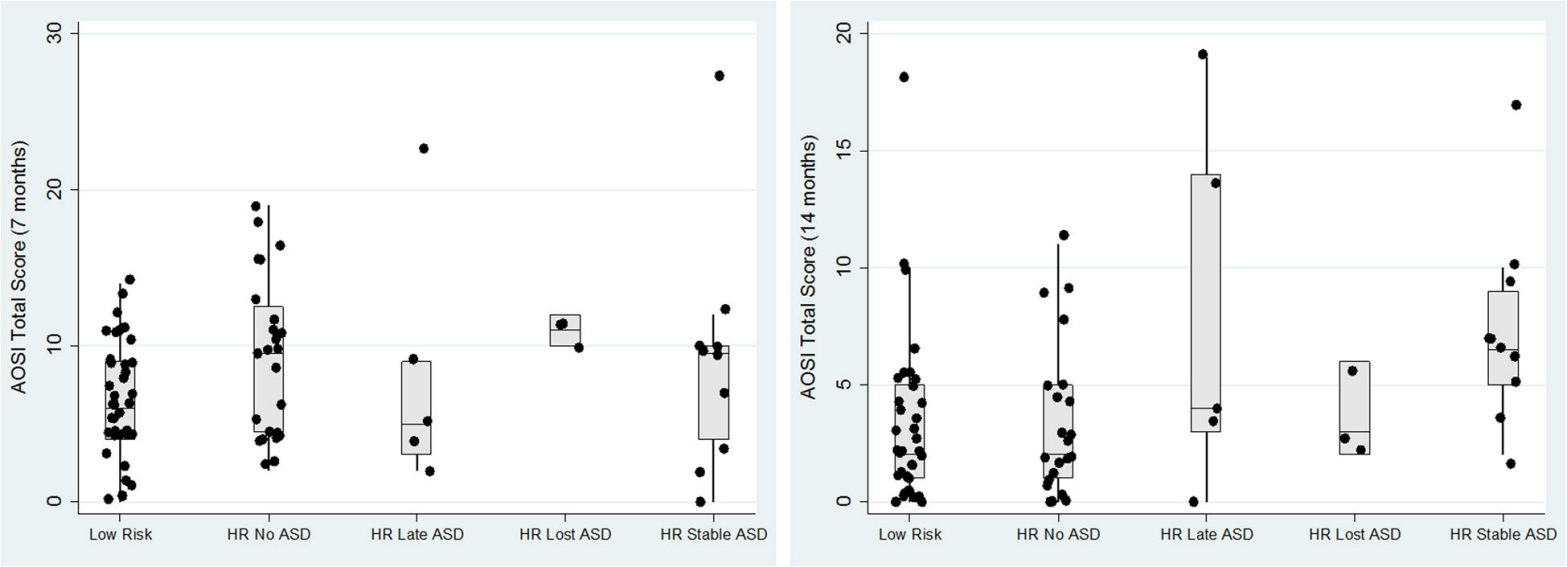
**a** AOSI total score at 7 months by stability of ASD outcome between 3 and 7 years. **b** AOSI total score at 14 months by stability of ASD outcome between 3 and 7 years

Due to the very low sample sizes of the ‘late diagnosis’ (*n* = 5) and ‘lost diagnosis’ (*n* = 3) groups, who had unstable patterns of meeting ASD diagnostic criteria across the 3- and 7-year assessments, it was not possible to statistically assess how neurocognitive and observational measures mapped on various trajectories in clinical manifestation. However, since neither the infant neurocognitive markers, P400 and disengagement, nor the AOSI have previously been examined in relation to such subgroups, we illustrate the distribution of scores on these measures in Fig. 3a and b and Fig. 4a and b. These distributions are suggestive of differential underlying mechanisms driving different profiles of ASD manifestation over early childhood.

## Discussion

This is the first study to demonstrate that infant antecedent markers, eye-gaze processing at 7 months and attention disengagement at 14 months, are associated with ASD diagnostic outcome in mid-childhood. Further, we show that at 7 months, P400 provides a better mapping onto mid-childhood ASD diagnosis than a concurrent observational measure of behavioural atypicality alone. Combining P400 with behavioural symptoms from the AOSI significantly improved the model fit compared to the AOSI alone, but the joint model did not improve prediction significantly compared to P400 alone. At 14 months, both neurocognitive and observational measures predicted ASD diagnosis in mid-childhood. A model incorporating both disengagement and 14-month AOSI was significantly better than having either AOSI alone or disengagement alone. Thus, at both infant ages, neurocognitive markers account for additional variation in the expression of later ASD outcome. However, at 14 months, the enhanced prediction from combining markers also emphasises that assessing a variety of measures of risk—including in this case *both* observational measures of early emerging ASD behaviours *and* experimental measures of neurocognitive function—in early infancy might remain the optimal approach to early screening. Multiplicative and additive models of ASD risk have been suggested [39], and certain infant neurocognitive markers have already been shown to predict ASD outcome in additive manner [40].

Although our results indicate that measuring specific neurocognitive systems may have as good, if not better, predictive power than comprehensive measures of behavioural atypicality, especially at an age when underlying ASD pathology has not yet exerted broader downstream effects on cognitive development, we are not yet able to advocate the use of ERPs or other neurocognitive phenotypes in ASD screening or diagnosis. Replication of both the infant neurocognitive markers and their association with 7-year outcome will be critical, as well as further validation steps such as test-retest reliability. Nevertheless, given the apparent added benefit of including the neurocognitive markers in addition to the AOSI, the next translational step may be to use these neurocognitive markers in triaging at-risk cases, initially identified on the basis of a screening tool or family risk.

Prediction from infant markers to mid-childhood outcome mirrored prediction of 3-year-old outcome (see Additional file 1). At both 3 and 7 years, the P400 differentiated HR-ASD and LR groups, but only marginally the HR-ASD and HR-No ASD groups. Similarly, for both the 3- and 7-year-old ASD outcome, attention disengagement differentiated HR-ASD from the other two groups. Similarity in prediction was found despite diagnostic change occurring between 3 and 7 years of age—with some of the children with a ‘late diagnosis’ joining the HR-ASD group (*n* = 5) and others, the ‘lost diagnosis’ (*n* = 3) leaving the group, at 7 years of age [26]. This may suggest that these two groups have similar profiles; perhaps children who meet criteria at just one time point have less severe symptoms and are closer to the diagnostic threshold. Although sample sizes prevented us from assessing group differences statistically, the descriptive statistics do not support the late and lost diagnosis groups having substantially less severe scores on the ADOS or the ADI compared to those with stable ASD (see Additional file 1: Table S1). Further, it is worth noting that the neurocognitive markers described in this paper showed differential group patterns across the ‘late’ and ‘lost’ diagnostic groups; while P400 effect showed lower (i.e. more atypical) values in both the late and stable ASD groups (i.e. those with a diagnosis at 7 years of age), poor disengagement mainly characterised the stable ASD group.

There is mounting evidence from twin studies that symptom stability over time indicates a high degree of genetic influence [41], possibly involving genes associated with neural plasticity [42]. Some markers might thus reflect the brain’s ability to adapt to early disturbance, delaying the onset of overt symptoms to later in childhood, while others might index highly penetrant traits leading to a stable expression of ASD symptoms throughout childhood. In terms of translational opportunities, understanding more about the underlying neurocognitive developmental processes that lead to different trajectories of phenotypic expression may also provide unique windows of opportunity for early detection and screening [43] or targeting pre-emptive interventions according to different patterns of early neurocognitive and behavioural manifestations [44, 45]. Larger samples will be required to test whether there are different patterns of early antecedent marker prediction of longitudinal trajectories of ASD symptoms, whether considered in dimensional or categorical (diagnostic) terms, and this is an important goal of future work.

### Limitations

Although 7 year diagnosis was not directly yoked to the diagnostic view reached at 3 years (as evident from the instability we report, see [26] for discussion), one significant limitation of the current study is the lack of independence between the 3 year and 7 year diagnostic judgements in that some of the clinical researchers were involved in both. It is also worth noting that the AOSI is the only measure of early ASD behaviours available from this cohort at the same time-point as the neurocognitive markers, whereas the neurocognitive markers reported in the main text of the paper are those ‘pre-selected’ as significantly related to 3-year ASD outcome in this sample. Replication in independent samples using a pre-defined analysis plan is thus necessary, and we are currently attempting to replicate the current findings in a separate cohort of children. Finally, as with all research utilising the familial high-risk sibling design, the generalisability to the wider population of children with ASD who are not from multiplex families cannot be assumed [46].

## Conclusion

While the focus of most of the work on predictive infant neurocognitive markers of ASD conducted to date has been on predicting diagnostic outcome at 2 or 3 years of age [10], the current study emphasises the value of continued follow-up of these cohorts through childhood. While future replication in independent samples is required, our results demonstrate the potential of antecedent markers indexing neurocognitive systems to improve early prediction of mid-childhood diagnosis of ASD. Further, such studies may help elucidate the neurodevelopmental mechanisms that underlie different developmental trajectories, as well as the environmental influences that affect developmental change and outcome in all children. In future, prospective longitudinal studies may uncover distinct neurodevelopmental mechanisms that lead from risk to disorder and highlight translational opportunities for screening and pre-emptive intervention.

## Additional file

Additional file 1: Supplementary materials. (DOCX 39 kb)

## Abbreviations

ADI-R: Autism Diagnostic Interview—Revised
ADOS-G: Autism Diagnostic Observational Schedule—Generic
AOSI: Autism Observation Scale for Infants
ASD: Autism Spectrum Disorder
BASIS: British Autism Study of Infant Siblings
CSS: Calibrated severity score
DAWBA: Development and Well-Being Assessment
DSM-5: Diagnostic and Statistical Manual 5th edition
HR: High risk
LR: Low risk
M-CHAT-R/F: Modified Checklist for Autism in Toddlers-Revised/Follow-Up
MSEL: Mullen Scales of Early Learning
SCQ: Social Communication Questionnaire
SRS-2: Social Responsiveness Scale—Second Edition
VABS-II: Vineland Adaptive Behavior Scales—Second Edition
WASI-II: Wechsler Abbreviated Scale of Intelligence—Second Edition

## Footnotes

1 5 DAWBA and 5 SCQ missing
