# Supplementary material for: Neurocognitive and observational markers: prediction of autism spectrum disorder from infancy to mid-childhood
Source: Mol Autism. 2017 Sep 22;8:49. doi: 10.1186/s13229-017-0167-3 (PMC5610446; doi:10.1186/s13229-017-0167-3)
Supplement: Supplementary file 1 — Supplementary materials. (DOCX 39 kb) [file 13229_2017_167_MOESM1_ESM.docx]

Supplementary information

*Supplementary methods*

*Stability of ASD diagnosis between age 3 and 7 years:* Forty-two HR and 37 LR participants were assessed for ASD at both 3 and 7 years of age. *At 3 years of age,* four experienced researchers (KH, SC, GP, TC) reviewed information across the research visits at 2 years (including ADOS-G, MSEL, and VABS-II assessments) and 3 years (including MSEL, VABS-II, ADOS-G and *Autism Diagnostic Interview – Revised*, *ADI-R*; Le Couteur, Lord & Rutter, 2003) and assigned clinical diagnoses of ASD according to ICD-10 (WHO, 1993). For the LR control children, no formal clinical diagnoses were made in the absence of a full developmental history (i.e., no ADI-R was collected) but none had a community clinical ASD diagnosis. The diagnostic process at the 7 year assessment is reported in the Methods of the main paper. For descriptive exploration of whether patterns of the infant antecedent markers might differ according to diagnostic stability vs. instability subgroups, HR children were categorised according to stability of diagnosis across the 3 and 7 year assessments. Thus, children who did not meet ASD criteria at either assessment were classified as HR-No-ASD (*n* = 24; 57%), children who met ASD criteria at both age 3 and age 7 years were assigned to a HR-Stable-ASD group (*n* = 10; 24%). Three children (7%) met ASD criteria only at age 3 but not at age 7, henceforth the ‘lost’ diagnosis group and 5 children (12%) met ASD criteria at 7 years but not at 3 years, henceforth the ‘late’ diagnosis group, see Table S1 for full details of ADI, ADOS and Mullen/WASI scores at the 3 and 7 year assessments.

*Supplementary Analyses*

*Associations with 3 year outcome in the subset of children with 7 year old follow-up*

When measured at 7 months, the AOSI total score was not a significant predictor of ASD outcome *χ^2^*(2) = 4.34, *p* = 0.11. At 14 months, however, AOSI score did significantly predict ASD outcome *χ^2^*(2) = 6.11, *p* = 0.047, with the HR-ASD group showing higher AOSI scores than LR controls (*B* = -0.193, *SE* = 0.081, odds ratio = 0.83, *p* = 0.018). The difference between HR-ASD and HR-No ASD was only marginal (*B* = -0.129, *SE* = 0.075, odds ratio = 0.88, *p* = 0.086).

Seven-month P400 amplitude difference score was a significant overall predictor of ASD outcome (*χ^2^*(2) = 9.93, *p* = 0.007), with the HR-ASD group differentiating less between the away and towards gaze shifts than LR controls (*B* = 0.258, *SE* = 0.098, odds ratio = 1.29, *p* = 0.008) but not than the HR-No ASD group (*B* = 0.129, *SE* = 0.086, odds ratio = 1.14, *p* = 0.13). Disengagement latency at 14 months also predicted overall ASD outcome (*χ^2^*(2) = 19.71, *p* < 0.001), with the HR-ASD group having significantly longer latencies than both other groups (LR: *B* = -0.018, *SE* = 0.006, odds ratio = 0.98, *p* = 0.002; HR-No ASD: *B* = -0.019, *SE* = 0.006, odds ratio = 0.98, *p* = 0.001).

*Additional biomarkers*

As well as the gaze ERP and disengagement measures in the main paper, we have previously published on an additional three antecedent biomarkers that associate with autism symptoms assessed at 3 years: fixation duration (Wass et al., 2015) and neural connectivity (Orekhova et al., 2014) and gaze following (Bedford et al., 2012). Sample size restrictions prevented us from analysing the connectivity measure and gaze following measures (<9 children in one of the groups). Fixation duration (weighted by number of fixations) was not related to 7-year-old ASD outcome: F(2, 65)=0.31, p=0.73. Thus the antecedent biomarkers reported in the main text of the paper, a neural measure of gaze processing and disengagement latencies, are those which were significantly related to autism outcome.

*Sex as a covariate*

Previous high-risk cohort studies revealed sex differences in the manifestation of early markers of ASD (Chawarska et al., 2016) or stronger prediction from early markers to 3-year-old ASD symptoms in boys than girls (Bedford et al., 2016). We re-ran the logistic regressions controlling for sex, but the results remained substantively similar. After controlling for sex, at 7 months the AOSI total score remained non-significant *χ^2^*(2) = 4.65, *p* = 0.098. At 14 months, AOSI score remained a significant predictor of ASD outcome *χ^2^*(2) = 10.33, *p* = 0.006, with the HR-ASD group showing higher AOSI scores than both LR controls and HR-No ASD (*p* values > 0.019). Seven-month P400 amplitude difference score was again a significant overall predictor of ASD outcome (*χ^2^*(2) = 12.41, *p* = 0.002), with the HR-ASD group differentiating less between the away and towards gaze shifts than LR controls (*B* = 0.288, *SE* = 0.10, odds ratio = 1.33, *p* = 0.004) and not reaching significance for the HR-No ASD group (*B* = 0.185, *SE* = 0.097, odds ratio = 1.20, *p* = 0.056). Disengagement latency at 14 months also remained a significant predictor of overall ASD outcome (*χ^2^*(2) = 8.64, *p* = 0.013), with the HR-ASD group having significantly longer latencies than both other groups (*p* values = 0.018).

*Removing one P400 outlier*

One child with autism had an outlying P400 difference score of -21.05. This value was trimmed back to -6.18 (just below the next lowest value of -6.17; Tabachnick & Fidell, 2007), and the analyses were re-run. Results remained substantively similar; 7-month P400 amplitude difference score was a significant overall predictor of ASD outcome (*χ^2^*(2) = 11.08, *p* = 0.004, with the HR-ASD group differentiating less between the away and towards gaze shifts than LR controls (*B* = 0.306, *SE* = 0.11, odds ratio = 1.36, *p* = 0.004) and the HR-No ASD group (*B* = 0.206, *SE* = 0.10, odds ratio = 1.23, *p* = 0.047). When the 7-month AOSI total score was included in the model (omnibus test *χ^2^*(4) = 13.92, *p* = 0.008), the overall effect of P400 remained significant (χ^2^(2) = 9.49, p = 0.009), while AOSI remained non-significant (χ^2^(2) = 2.84, p = 0.24). The P400 score significantly predicted the difference between HR-ASD and low risk controls (B = 0.289, SE = 0.11, odds ratio = 1.34, p = 0.006) and HR-No ASD (B = 0.21, SE = 0.11, odds ratio = 1.23, p = 0.045).

*Table S1* Descriptive statistics for the groups split by diagnostic change from 3 to 7 years of age

|  | **Low Risk**  **N = 37^*^** | **HR Never Diagnosed**  **N = 24^*^** | **HR Later**  **Diagnosis**  **N = 5^*^** | **HR Lost Diagnosis**  **N = 3** | **HR Stable Diagnosis**  **N = 10^*^** |
| --- | --- | --- | --- | --- | --- |
| *Sex (male: female)* | 15:22 | 5:19 | 2:3 | 3:0 | 5:5 |
| **3 year scores** |  |  |  |  |  |
| *ADI – Social* | Not completed | 2.75 (3.71) | 1.40 (1.95) | 5.67 (4.04) | 11.50 (4.12) |
| *ADI – Comm.* | Not completed | 2.96 (4.13) | 1.80 (1.64) | 3.67 (3.79) | 9.60 (5.06) |
| *ADI – RRB* | Not completed | 0.71 (1.20) | 0.80 (0.84) | 2.67 (2.08) | 3.70 (2.41) |
| *ADOS – CSS Total* | 3.17 (2.25) | 3.42 (2.54) | 3.60 (2.41) | 6.67 (0.58) | 5.90 (2.60) |
| *ADOS – SA CSS* | 3.92 (2.41) | 4.42 (2.77) | 4.80 (2.95) | 7.33 (0.58) | 6.10 (2.51) |
| *ADOS – RRB CSS* | 3.53 (2.57) | 4.04 (2.56) | 4.80 (2.28) | 7.00 (1.00) | 6.20 (2.39) |
| *SCQ* | 3.00 (2.64) | 3.75 (6.56) | 6.20 (5.07) | 5.33 (3.22) | 14.11 (7.93) |
| *SRS Preschool* | 42.00 (3.66) | 45.63 (10.60) | 49.80 (8.59) | 43.00 (3.61) | 62.67 (15.68) |
| *Mullen ELC* | 117.17 (15.76) | 109.83 (16.91) | 116.20 (18.21) | 99.00 (25.87) | 89.80 (27.86) |
| *Vineland ABC* | 105.91 (8.75) | 98.96 (10.24) | 104.40 (7.40) | 92.33 (4.16) | 90.40 (18.28) |
| **7 year scores** |  |  |  |  |  |
| *ADI – Social* | Not completed | 4.04 (5.48) | 11.60 (4.56) | 5.33 (2.31) | 14.00 (4.80) |
| *ADI – Comm.* | Not completed | 4.25 (4.67) | 10.60 (5.13) | 4.00 (1.73) | 10.33 (4.58) |
| *ADI – RRB* | Not completed | 0.58 (1.41) | 3.20 (.84) | 1.00 (1.00) | 3.78 (2.11) |
| *ADOS – Total CSS* | 1.70 (1.19) | 2.46 (1.41) | 6.40 (2.70) | 3.00 (1.73) | 6.10 (2.96) |
| *ADOS – SA CSS* | 2.18 (1.70) | 2.96 (1.60) | 7.00 (2.55) | 3.67 (2.08) | 6.40 (2.72) |
| *ADOS – RRB CSS* | 1.12 (0.70) | 3.04 (2.84) | 5.80 (2.95) | 3.33 (4.04) | 6.30 (2.71) |
| *SCQ* | 3.30 (6.53) | 2.64 (5.52) | 15.60 (13.43) | 3.33 (1.16) | 13.89 (9.51) |
| *SRS-2* | 45.49 (5.82) | 52.37 (11.74) | 80.00 (21.77) | 45.33 (3.79) | 71.63 (24.23) |
| *WASI FSIQ* | 117.06 (11.61) | 107.96 (12.76) | 111.60 (7.64) | 118.33 (18.01) | 108.78 (26.63) |
| *Vineland ABC* | 110.53 (6.98) | 102.22 (12.67) | 88.40 (14.55) | 116.33 (4.93) | 91.20 (16.57) |

**^*^**Group sizes are smaller for some variables due to missing data.

*References*

Bedford, R., Jones, E. J., Johnson, M. H., Pickles, A., Charman, T., & Gliga, T. (2016). Sex differences in the association between infant markers and later autistic traits. *Molecular autism*, *7*(1), 21.

Bedford, R., Elsabbagh, M., Gliga, T., Pickles, A., Senju, A., Charman, T., & Johnson, M. H. (2012). Precursors to social and communication difficulties in infants at-risk for autism: Gaze following and attentional engagement. *Journal of autism and developmental disorders*, *42*(10), 2208-2218.

Chawarska, K., Macari, S., Powell, K., DiNicola, L., & Shic, F. (2016). Enhanced Social Attention in Female Infant Siblings at Risk for Autism. *Journal of the American Academy of Child & Adolescent Psychiatry*, *55,* 188–195.e1

Le Couteur, A., Lord, C., & Rutter, M. (2003). *The Autism Diagnostic Interview-Revised (ADI-R).* Los Angeles, CA: Western Psychological Services.

Orekhova, E.V., Elsabbagh, M., Jones, E.J.H., Dawson, G., Charman, T., Johnson, M.H. and the BASIS Team. (2014). EEG hyper-connectivity in high-risk infants is associated with later autism. *Journal of Neurodevelopmental Disorders,* 6, 40.

Tabachnick, B.G., & Fidell, L.S. (2007). *Using multivariate statistics*. Boston MA: Pearson.

Wass, S. V., Jones, E. J., Gliga, T., Smith, T. J., Charman, T., & Johnson, M. H. (2015). Shorter spontaneous fixation durations in infants with later emerging autism. *Scientific reports*, *5*.

World Health Organisation. (1993). *Mental Disorders: A Glossary and Guide to their Classification in Accordance with the 10th Revision of the International Classification of Disease - Research Diagnostic Criteria: ICD-10*. Geneva: Author.
